# Functional Characterization of *GmRD22* Modulating Nitrogenase Activity in Soybean with Transcriptomic Comparison Between Two Genotypes

**DOI:** 10.3390/plants15152276

**Published:** 2026-07-25

**Authors:** Hanyu Zhao, Jiaying Zhong, Chao Ma, Tianhong Wang, Wenhao Fan, Qingbing Shi, Shengnan Ma, Chunshuang Tang, Lin Chen, Dawei Xin, Qingshan Chen, Chunyan Liu, Jinhui Wang

**Affiliations:** 1 National Key Laboratory of Smart Farm Technology and System, Key Laboratory of Soybean Biology in Chinese Ministry of Education, College of Agriculture, Northeast Agricultural University, Harbin 150030, China; 13592084681@163.com (H.Z.); 17530413833@163.com (J.Z.); mcneau@126.com (C.M.); 18845781550@163.com (T.W.); fan_wenhao060310@163.com (W.F.); 19846110860@163.com (Q.S.); dwxin@neau.edu.cn (D.X.); qschen@neau.edu (Q.C.); 2 Crop Development Research Institute, Heilongjiang Academy of Land Reclamation Sciences, Harbin 150038, China; mashengnan34@163.com (S.M.); 13644546046@163.com (C.T.); 3 Heilongjiang Academy of Agricultural Sciences, Harbin 150086, China; venguamecl@163.com

**Keywords:** soybean, symbiotic nitrogen fixation, RNA-Seq, *GmRD22*, functional study

## Abstract

Soybean (*Glycine max*) is a key crop in China grown as a source of edible oil and plant-derived protein, and its production is closely linked to national food security. Insufficient nitrogen availability remains a key constraint on soybean yield. In legumes, symbiotic nitrogen fixation (SNF) enables the conversion of atmospheric nitrogen into bioavailable forms, thereby reducing reliance on synthetic fertilizers and improving soil quality. Nitrogenase activity is a central determinant of SNF efficiency; however, its regulatory mechanisms in soybean nodules are not yet fully understood. In this study, transcriptomic data from two soybean accessions with contrasting SNF performance (Suinong 14 and ZYD00006) were analyzed, leading to the identification of *Glyma.04G013500* as a member of the *GmRD22* gene family. This study verified that this gene is potentially associated with nitrogenase activity. Functional characterization revealed that this gene acts as a negative regulator of nodulation by influencing the expression of genes associated with nodule development. Haplotype analysis further uncovered a pattern consistent with domestication, as the elite haplotype (HapI) with enhanced nitrogen fixation capacity, exhibited a progressive increase in frequency from wild soybean populations to landraces and modern cultivars. These findings suggest that *GmRD22* has undergone directional selection during soybean domestication and improvement. Overall, these results offer new insights into the genetic control of SNF and establish promising targets for breeding soybean varieties with improved nitrogen fixation efficiency.

## 1. Introduction

Soybean (*Glycine max* (L.) Merr.) is a globally important oilseed crop with substantial economic value and serves as a major source of high-quality plant protein. It contributes nearly one-quarter of the world’s plant-derived protein supply, underscoring its critical role in global food systems and agricultural sustainability [1,2]. Like other legumes, soybean plants establish a symbiotic relationship with compatible rhizobia, forming specialized root nodules that enable the biological fixation of atmospheric nitrogen (N_2_) through a process referred to as symbiotic nitrogen fixation (SNF) [3]. Nitrogen is indispensable for plant growth and yield formation, and nitrogen deficiency in soils is a major abiotic factor limiting agricultural productivity [4]. In soybean plants, SNF provides approximately 60–70% of the nitrogen required during the growing season, highlighting the importance of this symbiotic system for maintaining stable yields and enhancing soil fertility [5,6].

Over approximately 5000 years of domestication from wild soybean (*Glycine soja* Sieb. & Zucc.), many advantageous allelic variants associated with SNF efficiency have accumulated, forming a key genetic basis for adaptation and trait evolution [7,8]. Both natural selection and human-mediated breeding have progressively enriched germplasm resources with improved tolerance to low-nitrogen environments, facilitating coordinated improvements in yield and quality [9]. Despite advances in genomic technologies and breeding strategies, relatively few elite alleles directly regulating nitrogenase activity have been identified and functionally validated, indicating that additional key loci and regulatory mechanisms remain to be discovered [10,11,12].

Genes in the Responsive to Dehydration (*RD*) family serve as important regulators involved in plant responses to abiotic stress [13]. Their expression is controlled by complex signaling networks. Under dehydration conditions, disruption of cellular osmotic balance triggers a cascade of physiological and biochemical responses, including substantial changes in levels of hormones such as abscisic acid (ABA), which is important in regulating processes such as stomatal closure, thereby reducing water loss, and seed maturation and dormancy [14], in addition to activating *RD* gene expression through downstream signaling pathways involving MAPK cascades and AREB/ABF transcription factors, forming a core regulatory axis linking stress perception, ABA accumulation, and *RD* gene activation [15]. In addition to ABA-dependent pathways, *RD* genes can also be induced through ABA-independent mechanisms. For instance, environmental stresses such as low temperature and high salinity can directly activate *RD* gene transcription via transcription factors like DREB2, which bind to specific promoter elements [16]. This regulatory system enables flexible and rapid responses to diverse environmental challenges.

Although *RD* family genes share a common role in stress response, they are not confined to a single gene family but instead display substantial diversity. Based on sequence homology and conserved domains, *RD* genes are categorized into multiple families, including those encoding Late Embryogenesis Abundant (LEA) proteins, DREB transcription factors, and MYB transcription factors [16,17,18]. Members of different families contribute to stress tolerance through distinct mechanisms. For example, LEA proteins stabilize macromolecules and maintain cellular osmotic balance, whereas the proteins encoded by transcription factor-type *RD* genes regulate downstream gene networks to amplify stress-responsive signaling [19].

Since *RD* genes were first identified in 1992 [20], accumulating evidence has confirmed that typical *RD* family members such as *RD22* and *RD29* act as key responsive genes in plant abiotic stress adaptation. Both genes are rapidly upregulated under drought, salt and cold stress, and their ectopic overexpression significantly improves plant stress resistance [21]. For example, Arabidopsis *RD29A* is strongly induced by drought and cold, and boosts drought tolerance by promoting proline and osmoprotectant biosynthesis; rice *OsRD22* positively regulates salt tolerance, whereas *RD29B* mediates cold responses via an ABA-dependent signaling pathway [22]. Collectively, existing studies have almost exclusively linked the *RD22* gene family to drought and salt stress responses [23,24], with no reports addressing whether *RD22* participates in symbiotic nitrogen fixation (SNF).

SNF is a critical adaptive trait that allows soybean to survive nitrogen-poor soils, and nitrogenase activity is a direct physiological marker reflecting SNF efficiency [25]. Despite well-defined functions of *RD22* in abiotic stress, it remains unknown whether *RD22* family members modulate soybean nodulation and nitrogen fixation processes. In this study, we provide experimental evidence suggesting that *GmRD22-3*, one soybean *RD22* family member, may contribute to the regulation of soybean SNF efficiency by modulating nitrogenase activity, addressing a largely unexplored research question in this field. Population genetic analysis further suggests that *GmRD22-3* underwent artificial selection during soybean domestication, and its elite haplotype exhibits geographical distribution correlated with ecological adaptation. Our work expands the current understanding of *RD22* functions beyond canonical abiotic stress regulation, supplements existing knowledge of the regulatory network underlying soybean symbiotic nitrogen fixation, and offers a potential gene candidate for future breeding of high-nitrogen-fixation soybean varieties adapted to low-nitrogen agricultural soils.

## 2. Results

### 2.1. Marked Variation in Nitrogenase Activity Between Suinong 14 and ZYD00006 Reveals Divergence in Symbiotic Nitrogen Fixation Traits

**Figure 1:**
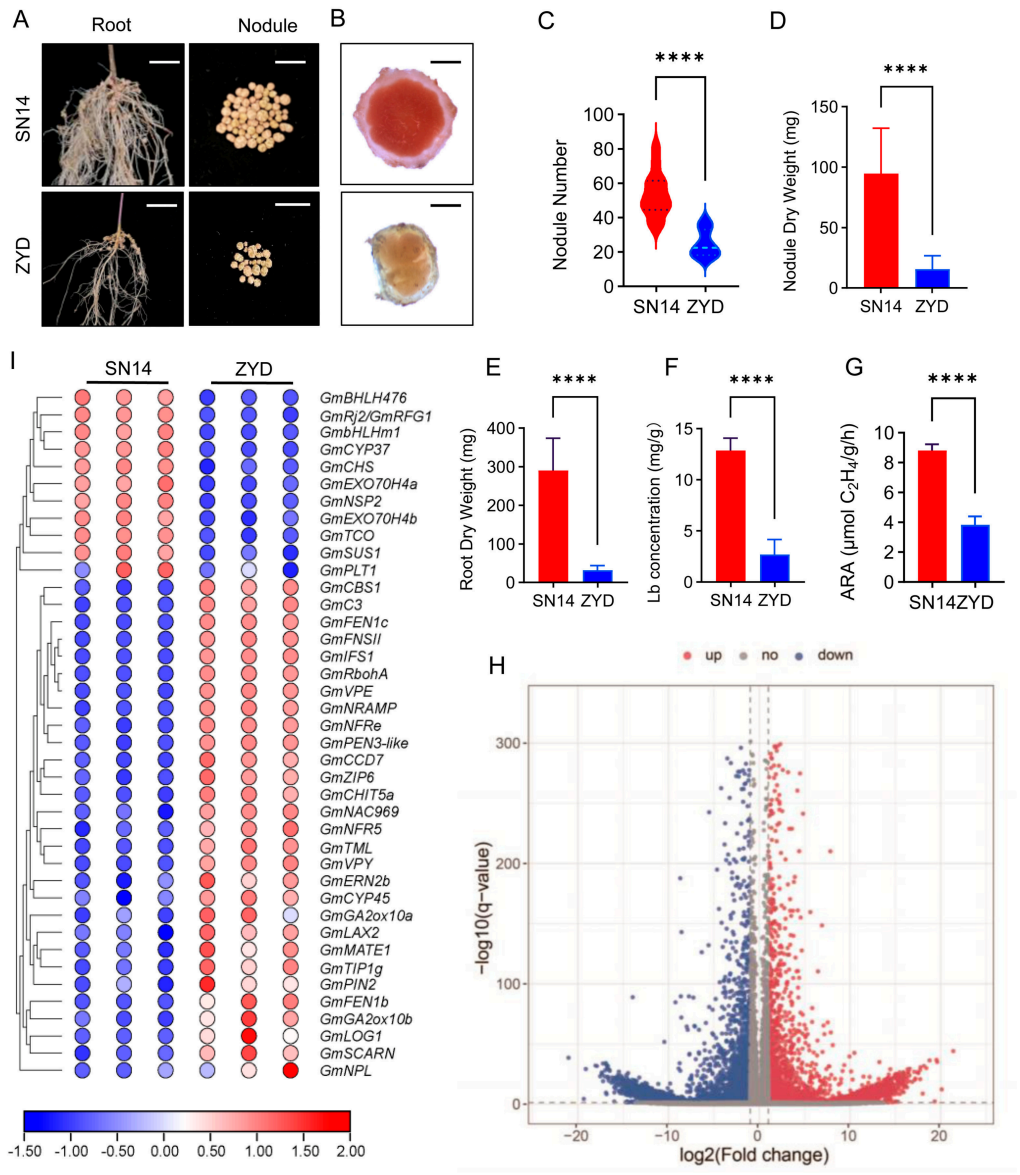
Differences in nodulation phenotypes and transcriptomic profiles between SN14 and ZYD. (**A**–**G**) Comparison of nodulation-related traits following rhizobial inoculation (root scale bars = 1 cm; nodule scale bars = 5 mm). Data represent mean ± SE (Standard Error) (n = 3 biological replicates); **** *p* < 0.0001 (Student’s t-test). (**B**) Cross-sectional images of mature nodules at 28 dpi (scale bars = 1 mm). (**H**) Volcano plot of DEGs between SN14 and ZYD nodules. The log_2_ (Fold Change) values are shown on the X-axis, with −log_10_(*p*-value) values on the Y-axis. Red and blue dots respectively correspond to genes that were significantly up- and down regulated (|log_2_FC| ≥ 1, *p* < 0.05), while gray dots correspond to genes that were not significantly differentially expressed. (**I**) Expression profiles of selected SNF-related marker genes differentially expressed between SN14 and ZYD.

To comprehensively compare SNF characteristics between cultivated and wild soybean, a controlled greenhouse pot experiment was conducted using the cultivar Suinong 14 (SN14) and the wild accession ZYD00006 (ZYD). Phenotypic evaluation demonstrated that SN14 consistently exhibited significantly higher values across multiple SNF-associated parameters, such as nodule number and dry weight, root dry weight, leghemoglobin content, and nitrogenase activity, indicating superior nitrogen fixation capacity relative to ZYD (Figure 1A–G). To investigate the molecular basis underlying these phenotypic differences, transcriptome sequencing was performed on root nodules collected at 28 days after rhizobial inoculation. Analysis of differential expression identified 6966 differentially expressed genes (DEGs) between SN14 and ZYD, with significant upregulation of 2916 and downregulation of 4050 in SN14 nodules (Figure 1H). Further examination of SNF-associated marker genes revealed 40 genes with significant expression differences, including 11 genes with higher expression in SN14 and 29 genes with higher expression in ZYD (Figure 1I).

The results GO analyses showed marked enrichment of DEGs (*p* < 10^−8^) in functional categories associated with membrane components, redox processes, transport activities, and immune-related responses. Specifically, enriched terms included oxidoreductase activity, monooxygenase activity, heme binding, iron ion binding, transmembrane transport, plasma membrane components, and defense responses (Supplementary Figure S1A). Further KEGG analyses demonstrated marked enrichment of DEGs in pathways associated with secondary metabolite biosynthesis (including phenylpropanoid, flavonoid, and isoflavonoid pathways), general metabolic processes, DNA replication, ABC transporters, plant hormone signal transduction, and circadian rhythm regulation. Among these, phenylpropanoid and flavonoid biosynthesis pathways showed the highest levels of enrichment, suggesting substantial differences between SN14 and ZYD in stress adaptation, defense mechanisms, and secondary metabolite accumulation (Supplementary Figure S1B).

### 2.2. Promoter Cis-Acting Element Analysis of the GmRD22 Gene Family

Members of the soybean *RD22* gene family were identified by BLAST searches against the Phytozome v12.1 database, using the conserved BURP domain sequence from *Arabidopsis thaliana AtRD22* (*AT5G25610*) as a query. This analysis identified 20 homologs in the soybean genome, defined as *GmRD22-1* to *GmRD22-20* (Table 1). The encoded proteins varied between 165 and 631 amino acid residues in length, with predicted isoelectric points ranging from 5.80 to 9.03 and molecular weights between 18.34 and 69.31 kDa (Table 1).

**Figure 2:**
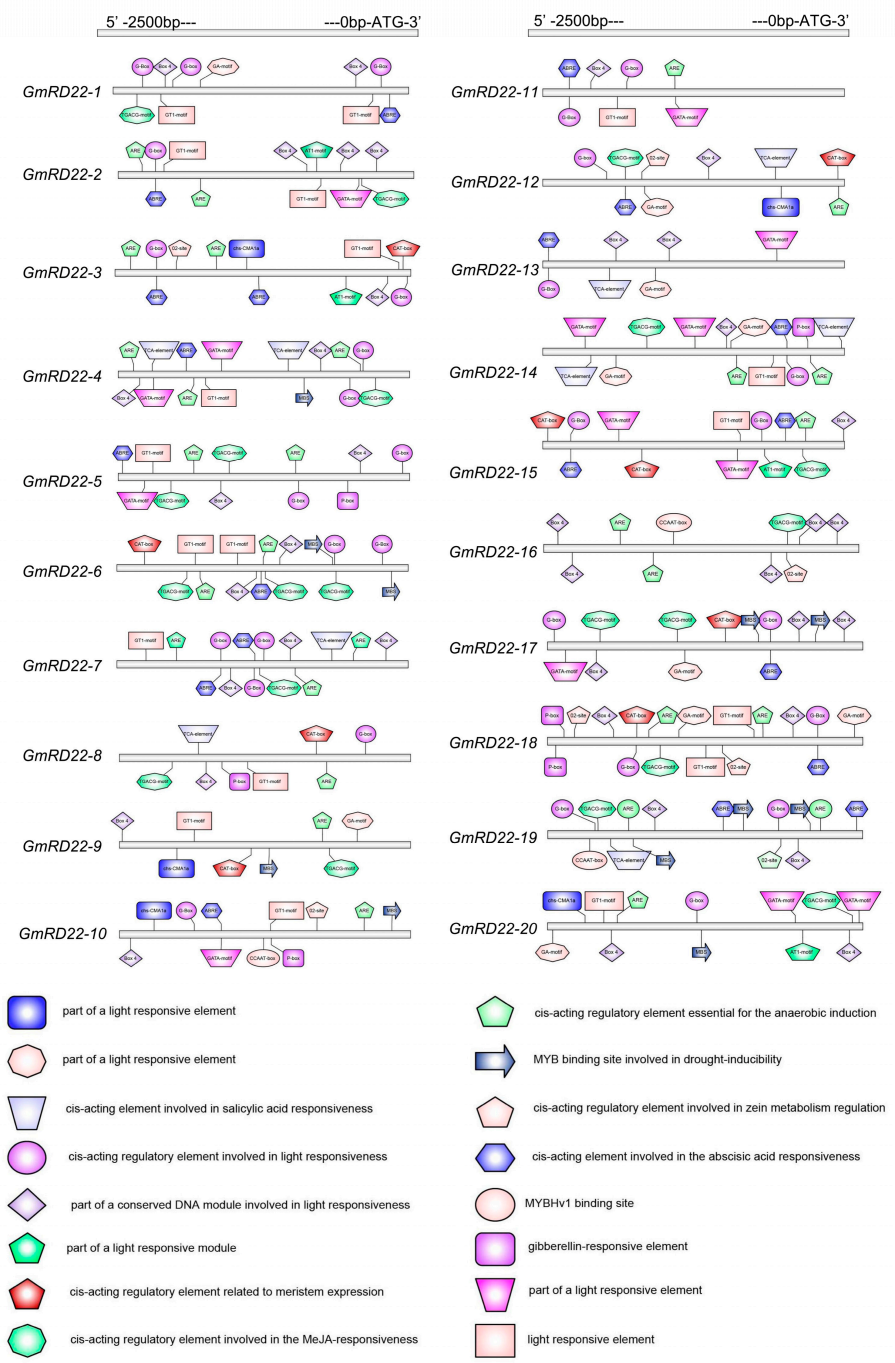
Predicted cis-acting regulatory elements in *GmRD22* promoter regions.

To explore potential regulatory mechanisms governing *GmRD22* gene expression, cis-acting elements within their promoter regions were analyzed. As illustrated in Figure 2, most *GmRD22* promoters contained numerous light-responsive elements, such as chs-CMA1a, GA-motif, G-box, Box 4, ARE, GATA-motif, and GT1-motif. In addition, a wide range of hormone-responsive elements was identified, including those responsive to abscisic acid (ABRE), salicylic acid (TCA element), methyl jasmonate (CGTCA motif), and gibberellins. Several genes, including *GmRD22*-*3*, *10*, *12*, *16*, and *18*, were found to contain the O2-site, a regulatory element associated with zein metabolism. Moreover, most *GmRD22* promoters harbored the CAT-box, which is linked to meristem-specific expression. Anaerobic response elements (ARE) were present in the majority of genes, except for *GmRD22*-*8*, *9*, *16*, and *20*, all *GmRD22*. Drought-responsive MYB-binding sites (such as CCAAT-box and MBS) were identified in *GmRD22*-*4*, *6*, *9*, *10*, *16*, *17*, *19*, and *20*. In addition, Gm*RD22*-*2*, *3*, *15*, and *20* contained the AT1-motif, which is associated with anaerobic induction. Overall, these promoter analyses suggest that *GmRD22* genes are likely involved in a range of biological processes, including light signaling, hormone-mediated regulation, stress responses, and developmental pathways.

### 2.3. Expression Pattern Analysis of the GmRD22s Gene Family

**Figure 3:**
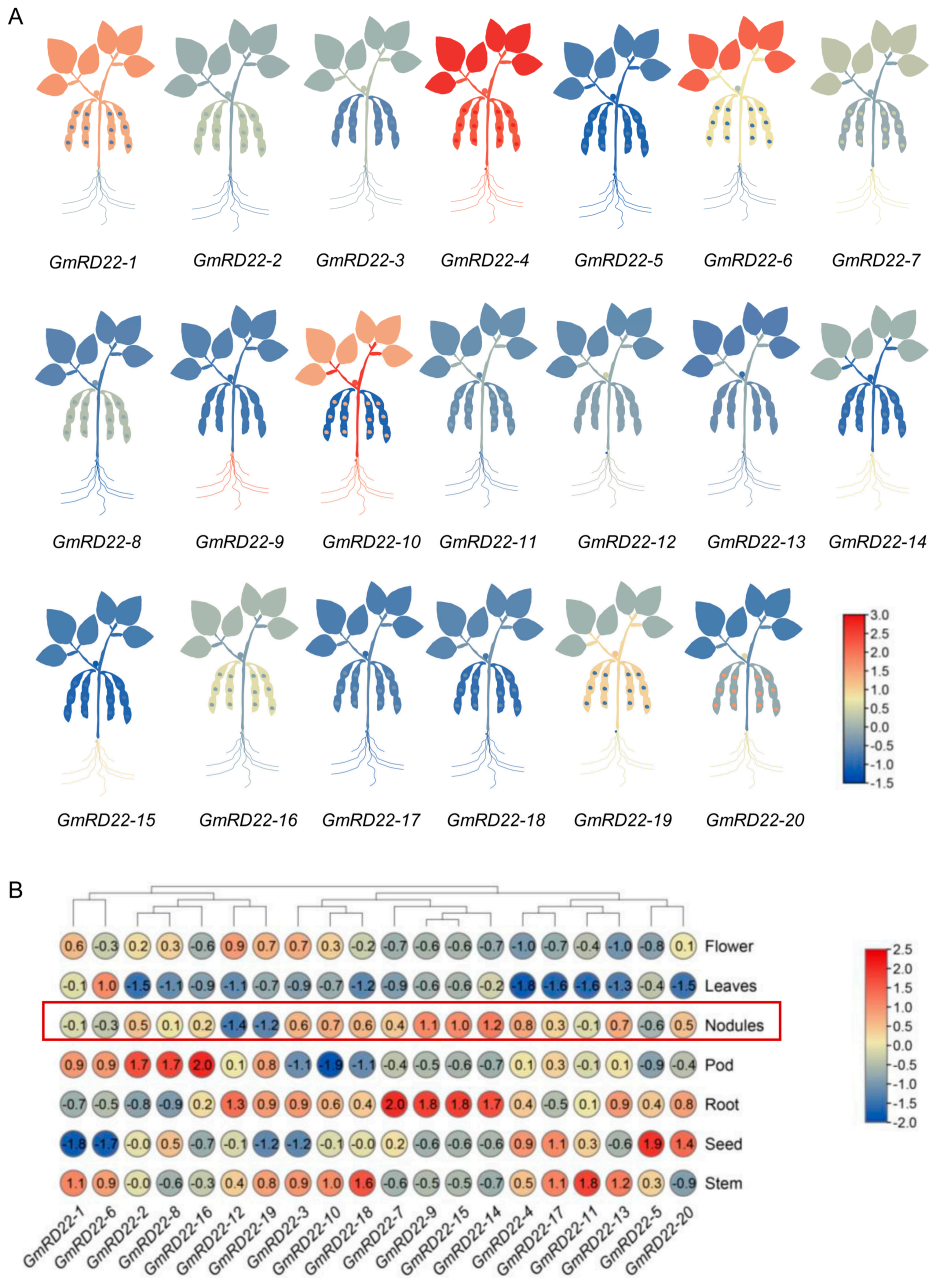
Expression patterns of *GmRD22* genes across different tissues. (**A**) Visualization of tissue expression of 20 GmRD22 genes in soybean whole plants. Color gradient indicates relative expression levels, with red representing high expression and blue representing low expression. (**B**) Heatmap of log-transformed expression values of GmRD22 genes in different tissues including flower, leaf, nodule, pod and root. The red box highlights the nodule tissue, which is the core tissue for nodulation-related functional analysis in this study.

To examine the spatial expression patterns of *GmRD22* genes, transcriptomic data from the Phytozome 14 database were analyzed across multiple soybean tissues. The results indicated that *GmRD22* family members are broadly expressed in leaves, roots, flowers, pods, nodules, shoot apices, and seeds (Figure 3A). Genes exhibiting expression levels greater than 0.5 in nodules were selected as candidates for further analysis, including *GmRD22-2*, *GmRD22-10*, *GmRD22-18*, *GmRD22-9*, *GmRD22-15*, *GmRD22-14*, *GmRD22-4*, *GmRD22-13*, and *GmRD22-20* (Figure 3B).

### 2.4. Candidate GmRD22 Gene Identification

**Figure 4:**
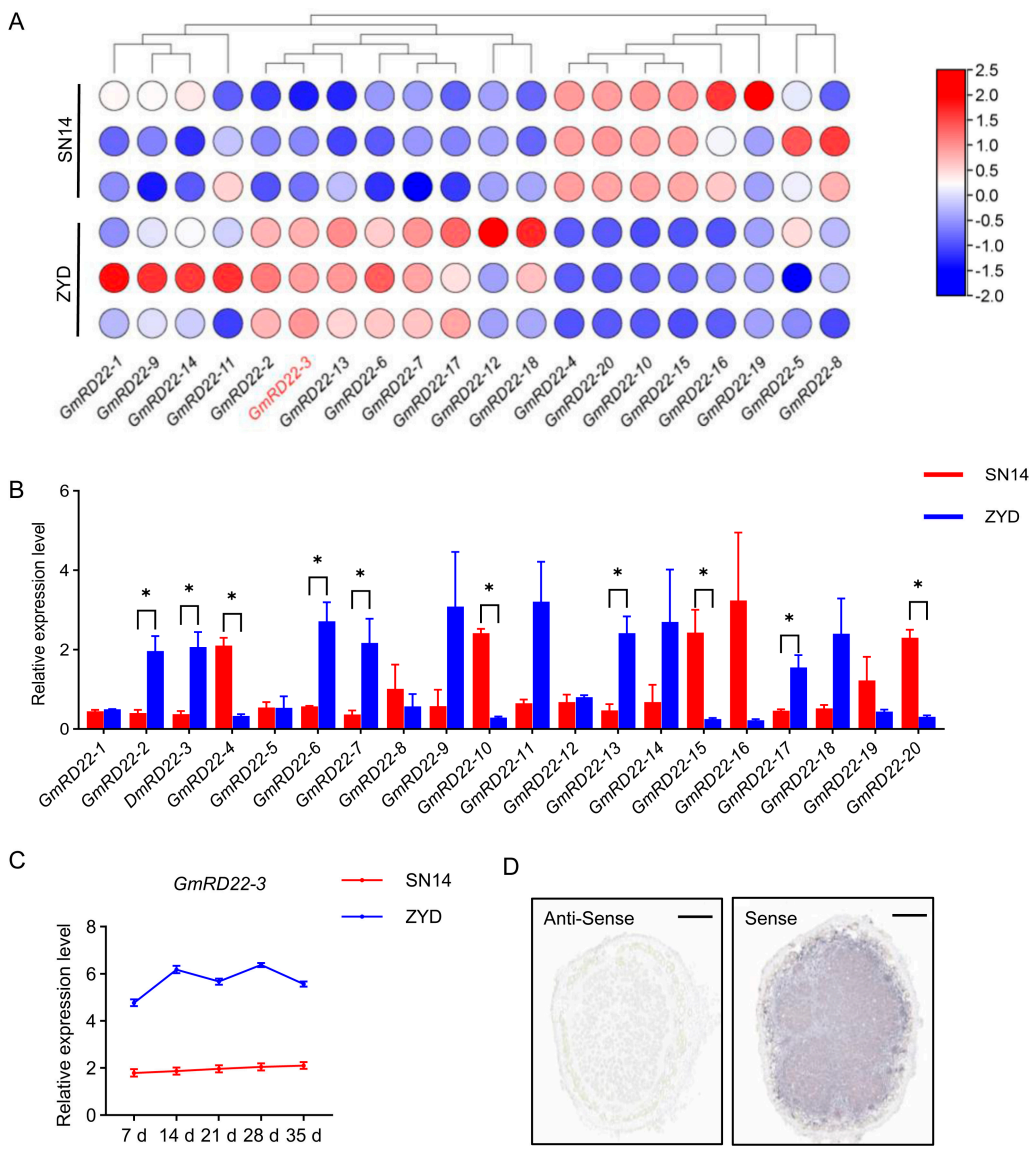
Expression of GmRD22 genes in SN14 and ZYD and spatiotemporal characterization of *GmRD22-3*. (**A**) Heatmap illustrating transcriptional patterns of *GmRD22* family members in nodules of SN14 and ZYD. (**B**) qRT-PCR validation of differentially expressed *GmRD22* genes. Relative transcript abundance of selected family members in nodules of SN14 and ZYD is shown as mean ± SD (n = 3 biological replicates).* *p* < 0.1 (Student’s t-test). (**C**) Time-course expression analysis of *GmRD22-3* in soybean roots. Transcript levels in SN14 and ZYD were measured at specified time points following inoculation (0, 7, 14, 21, and 28 dpi). (**D**) Spatial distribution of *GmRD22-3* transcripts in nodules. RNA in situ hybridization was used to visualize gene expression in SN14 and ZYD nodules. Scale bars = 500 μm.

To systematically evaluate the functions of *GmRD22* family members during soybean nodule development, transcriptomic profiling combined with quantitative expression analysis was conducted using two representative genotypes, SN14 and ZYD. A total of 10 *GmRD22* genes displayed significant differential expression in nodules. Based on expression specificity and potential functional relevance, seven genes were selected as key candidates for further investigation, including *GmRD22-2*, *GmRD22-3*, *GmRD22-13*, *GmRD22-4*, *GmRD22-20*, *GmRD22-10*, and *GmRD22-15* (Figure 4A,B).

To characterize their temporal expression dynamics, qRT-PCR analysis was performed at five stages in development (7, 14, 21, 28, and 35 days after inoculation). The results revealed distinct expression patterns among these genes. Specifically, *GmRD22-2*, *GmRD22-13*, *GmRD22-4*, *GmRD22-20*, *GmRD22-10*, and *GmRD22-15* exhibited stage-dependent or fluctuating expression profiles in both SN14 and ZYD (Supplementary Figure S2A–F). In contrast, *GmRD22-3* presented with consistently higher expression in ZYD across all examined stages compared with SN14 (Figure 4C), and its overall expression level exceeded that of the other candidate genes. This stable and elevated expression pattern suggests that *GmRD22-3* may play a sustained and central role in regulating nodule initiation, development, or maturation. Accordingly, *GmRD22-3* was prioritized for subsequent functional analysis. To further investigate its spatial distribution, RNA in situ hybridization was performed on SN14 nodules at 28 days post-inoculation. The results demonstrated strong *GmRD22-3* expression in both infected cells and the endodermal region of nodules (Figure 4C,D), consistent with qRT-PCR findings. This localization pattern suggests a close association between *GmRD22-3* expression and nitrogenase activity within nodular tissues. Subcellular localization analysis revealed that *GmRD22-3* is localized to the nucleus (Supplementary Figure S3). We conducted phylogenetic and interspecific synteny analyses to preliminarily explore the evolutionary features of the *GmRD22* family (Supplementary Figure S4A,B). The 20 soybean *GmRD22* proteins and *RD22* homologs from legumes and Arabidopsis thaliana formed separate branches with moderate node support, hinting at conserved divergence of the *RD22* family within legumes; nevertheless, limited taxon sampling weakens the reliability of broad evolutionary deductions for the whole gene family. Cross-species synteny detection identified collinear blocks containing *RD22* loci among various legumes, which offers tentative evolutionary hints regarding the nodule nitrogenase regulatory function of *GmRD22-3* characterized in our functional assays, though such inferences remain preliminary.

### 2.5. GmRD22-3 Negatively Regulates Nitrogenase Activity During Soybean Symbiotic Nodulation

**Figure 5:**
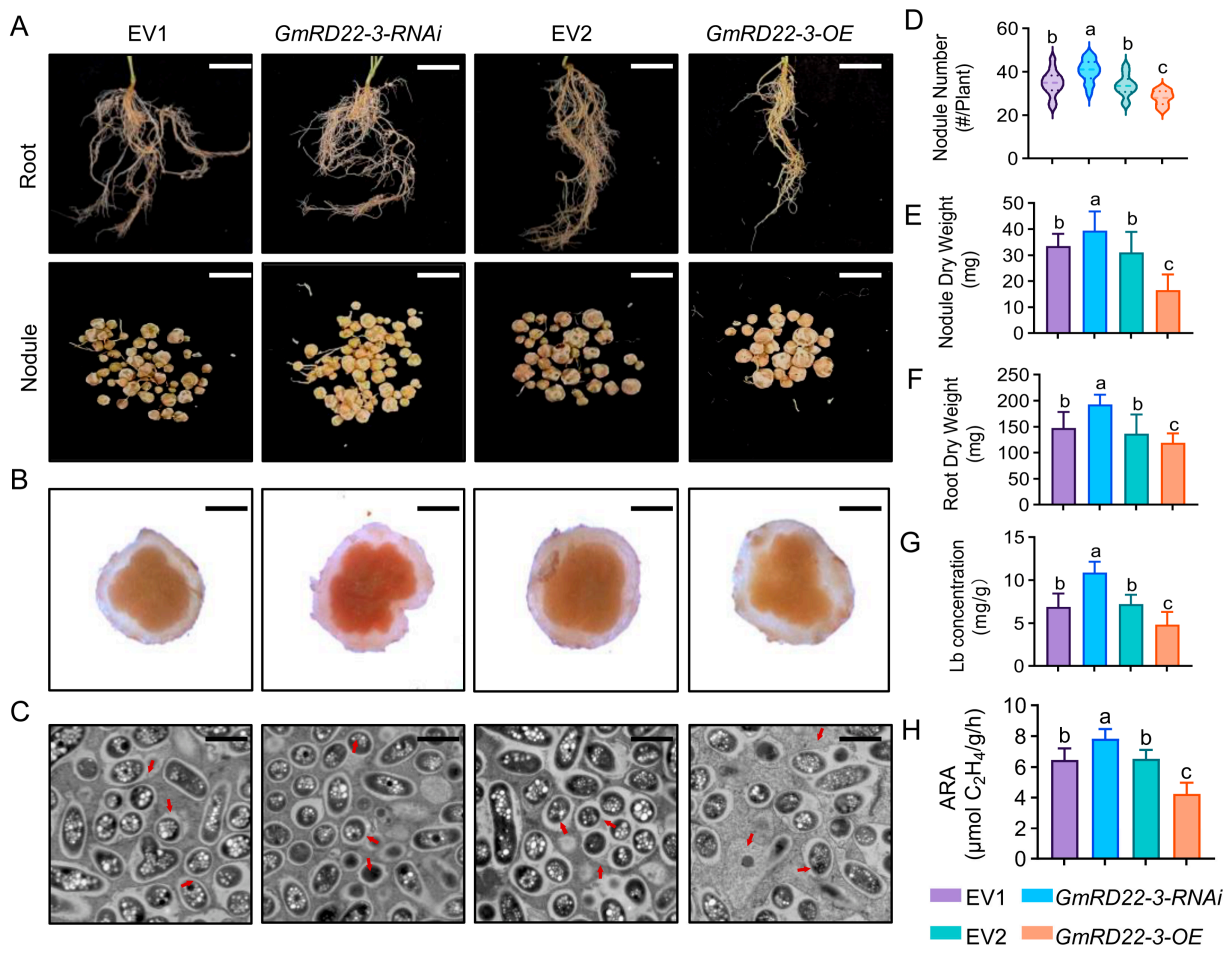
GmRD22-3 negatively regulates nitrogenase activity during soybean symbiotic nodulation. (**A**) Representative nodulation phenotypes of soybean plants expressing *GmRD22-3*-RNAi (gene silencing) or *GmRD22-3*-OE (overexpression) constructs, compared with empty vector (EV) controls. Scale bars: 5 cm (shoots) and 5 mm (roots). (**B**) Histological cross-sections of mature nodules from EV, *GmRD22-3*-RNAi, and *GmRD22-3*-OE lines at 28 days post-inoculation (dpi). Scale bars = 1 mm. (**C**) Ultrastructural analysis of symbiosomes in infected nodule cells by transmission electron microscopy (TEM) at 28 dpi. V, lytic vacuole; S, symbiosome. Scale bars = 1 μm. (**D**) Quantification of nodule number per plant. Values are presented as mean ± SD (n = 15 biological replicates). (**E**) Measurement of nodule fresh weight and shoot dry weight per plant. Data are shown as mean ± SD (n = 15 biological replicates). (**F**) Determination of leghemoglobin (Lb) content and nitrogenase activity in nodules. Values are expressed as mean ± SD (n = 15 biological replicates). One-way ANOVA followed by Tukey’s multiple comparison test was used for statistical analysis in panels (**D**–**F**); different lowercase letters indicate significant differences at *p* < 0.05. (**G**) Leghemoglobin (Lb) content in. (**H**) Analysis of the nitrogenase activity in.

To investigate the functional role of *GmRD22-3*, soybean carrying transgenic hairy roots in which this gene was knocked down (*GmRD22-3*-RNAi) or overexpressed (*GmRD22-3*-OE) were generated in the SN14 background (Figure 5A; Supplementary Figure S5A). All functional phenotypes below were exclusively obtained from this transient hairy root composite plant system, which has inherent chimeric limitations and may not fully reflect physiological performance of stably transformed whole soybean plants. Phenotypic analyses demonstrated a clear negative correlation between *GmRD22-3* expression and nodulation-related traits within transgenic hairy roots. Silencing *GmRD22-3* significantly increased key indicators of nitrogen fixation, including nodule number, nodule biomass, root dry weight, leghemoglobin content, and nitrogenase activity (Figure 5D–H), compared with the control. In contrast, overexpression lines exhibited significant reductions in all these parameters, implying that *GmRD22-3* acts as a negative regulator of nitrogen fixation efficiency in hairy root nodules. Further morphological and cytological analyses provided additional insight into its functional role. At 28 days post-inoculation, nodules from *GmRD22-3*-RNAi plants retained a characteristic pink coloration, reflecting high leghemoglobin content and active physiological status. In contrast, nodules from *GmRD22-3*-OE plants appeared visibly paler, consistent with premature senescence (Figure 5B). Transmission electron microscopy (TEM) revealed pronounced ultrastructural differences. In *GmRD22-3*-OE nodules, infected cells displayed extensive accumulation of poly-β-hydroxybutyrate (PHB) granules, along with disruption and degradation of symbiosome membranes, indicative of accelerated senescence and reduced bacteroid activity. In contrast, *GmRD22-3-RNAi* nodules showed minimal PHB deposition, with intact symbiosome structures and well-organized bacteroids, consistent with sustained nitrogenase function (Figure 5C). Together, these results from composite hairy root materials illustrate the involvement of *GmRD22-3* in modulating nodulation and nitrogen fixation under this transient experimental setup. Reduced expression delays nodule senescence and maintains nitrogenase activity, whereas elevated expression accelerates nodule aging and compromises nitrogen fixation capacity in transgenic hairy roots.

### 2.6. Haplotype Characterization of GmRD22-3

**Figure 6:**
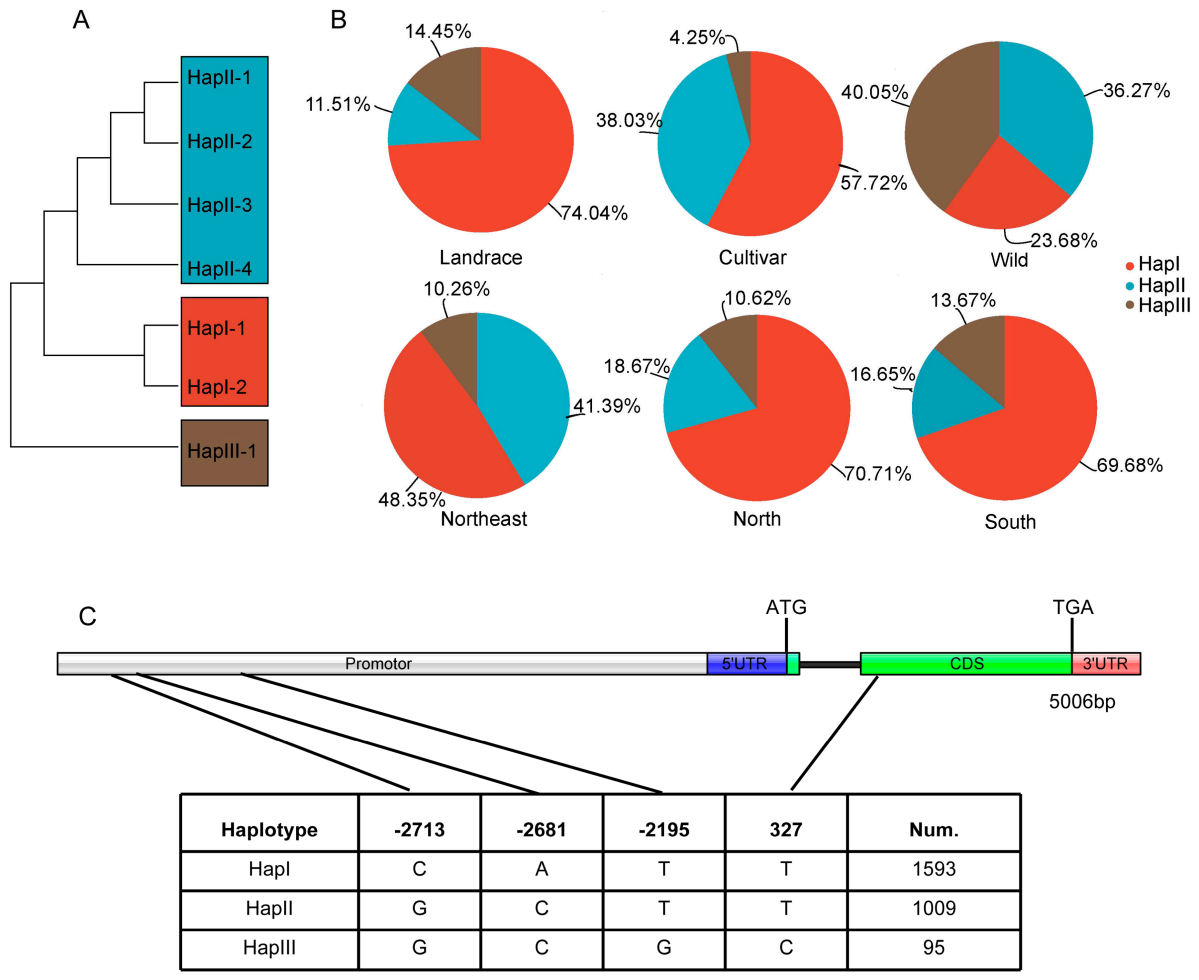
Haplotype analysis of *GmRD22-3*. (**A**) Phylogenetic relationships among haplotypes. (**B**) Geographic distribution and classification of soybean accessions carrying distinct haplotype, showing the proportions of each haplotype across accessions and regions. Wild: wild soybean (*Glycine soja*). Northeast: Northeast China; North: Huang–Huai–Hai region; South: Southern China. (**C**) SNP variations in *GmRD22-3* among different haplotypes.

To investigate the evolutionary dynamics of *GmRD22-3* over the course of soybean domestication and geographic diversification, we first conducted analyses of selection signatures and domestication history. These results tentatively indicated that *GmRD22-3* has undergone artificial selection following soybean domestication (Supplementary Figure S5B). Phylogenetic reconstruction of haplotypes classified all variants into three distinct groups: HapI, HapII, and HapIII (Figure 6A). We identified three haplotypes of *GmRD22-3* (Figure 6C), among which Hap1 and Hap2 were the predominant haplotypes. A total of four significant SNP sites were detected in both the promoter and CDS regions of *GmRD22-3*. The core differential SNP sites distinguishing HapI, HapII, and HapIII were mainly distributed in the promoter region rather than the coding region of *GmRD22-3*. Analysis of haplotype frequencies revealed a clear domestication-associated pattern. Specifically, the proportion of the elite haplotype HapI increased progressively from wild soybean populations to landraces and further to modern cultivars (Figure 6B), potentially suggesting strong positive selection during breeding. In addition, geographic distribution analysis showed that HapI occurred most frequently in the Huang–Huai–Hai region and least frequently in Northeast China, forming a pronounced latitudinal gradient. This spatial pattern may imply that the distribution of HapI is closely linked to environmental and ecological selection pressures (Figure 6B).

## 3. Discussion

The present study provides new insight into the functional role of *GmRD22-3* and highlights the structural and functional diversity within the *RD* gene group. Previous research has established that *RD* family genes do not constitute a single homologous gene family but instead represent a functionally defined group of genes distributed across multiple gene families [20]. Although all *RD* genes are unified by their involvement in dehydration stress responses, they exhibit low sequence similarity due to their diverse evolutionary origins. Our phylogenetic analysis of *RD22* homologs across angiosperms further supports this divergent evolutionary pattern (Supplementary Figure S4A) In *A. thaliana*, only a single *RD22* gene has been identified [26,27], consistent with prior genomic annotations and functional analyses [28,29], underscoring the conserved yet distinct nature of this gene in that species. Interspecific synteny analysis revealed limited collinear blocks of *RD22* loci between soybean and Arabidopsis (Supplementary Figure S4B), which offers evolutionary evidence to explain why *GmRD22-3* evolved unique symbiotic regulatory functions absent from the Arabidopsis ortholog.

Members of the BURP domain-containing protein family are known to participate broadly in plant physiological processes [30], including growth regulation, stress adaptation, and metabolic control [31,32]. For instance, certain BURP proteins contribute to seed development [33], enhance tolerance to abiotic stresses [34], and mediate cross-talk between biotic and abiotic stress responses. *RD22* is a representative BURP family member that has been linked to drought and salinity stress responses [33]. However, the present findings suggest that *RD22* may also participate in the regulation of SNF in soybean. Combining our promoter cis-element profiling and hairy root functional phenotypes, we identified abundant nodulation, hypoxia and senescence-related motifs in the 2500 bp upstream regulatory region of *GmRD22-3*, which could provide a potential cis-regulatory basis for its nodule-biased expression and function. These results therefore extend the known functional repertoire of the BURP family and offer additional insight into functional divergence among *RD* gene members, while also emphasizing the broader biological roles of the BURP domain [35]. Interestingly, bioinformatic prediction initially localized *GmRD22-3* to chloroplasts/plastids, which conflicted with the cytoplasmic symbiosome environment of symbiotic nitrogen fixation. This inconsistency highlights the inherent limitations of automated subcellular localization prediction tools relying solely on sequence features without in vivo experimental validation. We verified its subcellular localization and confirmed that *GmRD22-3* targets the cell nucleus, revealing the limitations of purely bioinformatic prediction. As a nuclear protein, *GmRD22-3* can regulate the transcription of nitrogen fixation-related genes in nodules, underlying its negative regulatory role in soybean symbiotic nitrogen fixation observed in our hairy root and TEM assays.

Consistent with transcriptomic data, *GmRD22-3* showed obvious genotypic expression differences in soybean nodules, suggesting its key role in nodule development and nitrogen fixation. Functional assays with transgenic hairy roots suggest that *GmRD22-3* may primarily modulate nodule senescence within this transient root system. Combined transcriptomic and phenotypic evidence indicates that *GmRD22-3* regulates soybean symbiotic nitrogen fixation primarily by mediating nodule senescence.

The haplotype analysis conducted in this study provides preliminary insights into the evolutionary trajectory of *GmRD22-3* and its adaptive significance. We observed a gradual enrichment of the elite haplotype HapI during domestication from wild soybean to landraces and modern cultivars. This observational pattern leads to a speculative hypothesis that this variant may imply a potential signature of directional selection. We further HapI may potentially harbor advantageous alleles associated with improved nitrogen use efficiency or adaptation to nitrogen-limited environments. which could have undergone weak positive selection and gradual enrichment during soybean domestication and breeding.

Analysis of geographic population structure revealed that HapI occurs at its highest frequency in the Huang–Huai–Hai region and at its lowest frequency in Northeast China, indicating a pronounced latitudinal cline. This spatial pattern shows a correlative association with the gradient of topsoil nitrogen levels across major soybean-producing areas in China. Specifically, soils in Northeast China are relatively nitrogen-rich and correspond to a low prevalence of HapI, whereas the Huang–Huai–Hai region, characterized by lower soil nitrogen availability, exhibits a markedly higher frequency of this haplotype. Notably, this distribution closely mirrors the haplotype pattern reported for the *GmNAR5* gene by Ma et al. [25], suggesting a possible shared adaptive pattern in response to soil nitrogen conditions. It is worth noting that these evolutionary and adaptive inferences are preliminarily concluded based on population genomic correlation evidence; direct functional validation is still required to further confirm the positive selection effect and adaptive advantage of HapI in low-nitrogen environments.

Functional evidence from our hairy root transformation assays further supports a role for *GmRD22-3* in nitrogen regulation. Specifically, hairy root-mediated transgenic overexpression of *GmRD22-3* accelerates nodule senescence and significantly suppresses nitrogenase activity, demonstrating that this gene acts as a negative regulator of symbiotic nitrogen fixation in soybean. The convergence in haplotype distribution between *GmRD22-3* and *GmNAR5* leads us to hypothesize that *GmRD22-3* may also contribute to adaptive responses under nitrogen-limited conditions. In particular, in low-nitrogen environments such as the Huang–Huai–Hai region, soybean accessions harboring HapI might potentially obtain a selective advantage by fine-tuning nodule development and optimizing nitrogen fixation efficiency.

Together, these findings provide new molecular insights into soybean domestication and ecological adaptation. They also suggest that *GmRD22-3* haplotypes may serve as promising candidate targets for marker-assisted selection, offering potential practical guidance for breeding soybean varieties with enhanced nitrogen fixation capacity and improved performance under low-nitrogen conditions.

Notably, this study has several inherent limitations that deserve further improvement. First, our phylogenetic and synteny analyses for the *GmRD22* gene family were based on limited species datasets and failed to present the homologous linkage between *GmRD22-3* and *GmRD22-7*, which may restrict the comprehensive interpretation of the family evolutionary trajectory. Second, all functional verification in this study relies on the hairy root transient transformation system. As a chimeric model, this system fails to fully recapitulate the stable genetic background of intact soybean plants and may bias assessments of the long-term regulatory effects of *GmRD22-3*. Accordingly, the observed regulatory patterns of *GmRD22-3* on nodulation and nitrogenase activity are limited to composite hairy root materials and cannot be directly extrapolated to stably transformed soybean lines. Third, the adaptive significance of different *GmRD22-3* haplotypes was inferred from population correlation analysis, lacking direct genetic or field experimental evidence. This study’s haplotype analysis is limited to statistical association analysis only. We did not conduct bioinformatic predictions to evaluate whether the defining SNPs alter transcription factor binding sites, cis-regulatory elements, or mRNA stability. Without such functional evidence, we cannot fully explain the molecular mechanism linking haplotype variation to the target phenotype. In addition, the specific molecular regulatory network, including the interaction partners and downstream pathways of *GmRD22-3*, remains unclear. Future studies will expand the phylogenetic dataset, adopt stable transgenic materials and field trials, and conduct molecular biochemical experiments to systematically clarify the evolutionary characteristics, biological functions and regulatory mechanisms of *GmRD22-3* and in vitro functional assays to resolve the above haplotype-related limitations.

## 4. Materials and Methods

### 4.1. Plant Materials, Rhizobia, and Growth Conditions

Soybean accessions SN14 and ZYD00006 were used as experimental materials. For nodulation assays, overnight exposure to chlorine gas was used to sterilize seeds, which were subsequently planted in sterilized double-pot systems. Seedlings were inoculated with rhizobial suspensions prepared in sterile water containing 10 mM MgSO_4_ at an optical density (OD_600_) of 0.2. Nodulation phenotypes were evaluated at 28 days after inoculation. The fast-growing rhizobium strain HH103 was used for inoculation and cultured in TY medium at 28 °C [36].

### 4.2. Transcriptomic Sequencing

Total RNA was isolated using TRIzol (Invitrogen, Carlsbad, CA, USA) as directed. RNA quality and integrity were evaluated using an Agilent Bioanalyzer 2100 (Agilent Technologies, Santa Clara, CA, USA) with the RNA 6000 Nano LabChip Kit (Agilent Technologies, Santa Clara, CA, USA), and only samples with an RNA integrity number (RIN) > 7.0 were retained for subsequent analyses. Three independent biological replicates were set for each group. For library preparation, 5 μg of total RNA was used for mRNA enrichment via two rounds of purification with Dynabeads Oligo (dT) (Invitrogen, Carlsbad, CA, USA). The purified mRNA was fragmented into short fragments using divalent cations at 94 °C for 5–7 min (Magnesium RNA Fragmentation Module, NEB (New England Biolabs, Ipswich, MA, USA)). First-strand cDNA was synthesized using SuperScript II Reverse Transcriptase, followed by second-strand synthesis using *E. coli* DNA polymerase I, RNase H, and dUTP to generate U-labeled double-stranded DNA. An adenine residue was added to the 3′ ends of the DNA fragments to enhance ligation of adapters. Dual-index adapters with thymine overhangs were ligated, and AMPure XP beads were utilized for selection of fragment sizes. Following treatment with heat-labile UDG enzyme to remove U-containing strands, the libraries underwent PCR amplification as follows: initial denaturation at 95 °C for 3 min; 8 cycles of 98 °C for 15 s, 60 °C for 15 s, and 72 °C for 30 s; and a final extension at 72 °C for 5 min. The resulting libraries had an average insert size of approximately 300 ± 50 bp. An Illumina NovaSeq 6000 platform (Illumina Inc., San Diego, CA, USA) was used for paired-end sequencing (2 × 150 bp).

Raw reads were quality-trimmed using Trimmomatic, and clean reads were mapped to soybean reference genome via HISAT2. Gene expression was normalized to TPM values. Differential expression analysis was performed on the LC-Bio Cloud Platform, with |log_2_(fold change)| ≥ 1 and FDR (Benjamini–Hochberg correction) < 0.05 as threshold for significant DEGs. Raw sequencing data are not deposited in public databases currently due to ongoing follow-up research, but can be provided upon reasonable request. All wet-lab library preparation and Illumina paired-end sequencing procedures were carried out strictly in accordance with LC-Bio’s standardized protocol for eukaryotic reference-guided transcriptome sequencing.

### 4.3. qRT-PCR

Nodule samples were harvested at 7, 14, 21, 28, and 35 days following rhizobial inoculation for gene expression analysis. Total RNA was isolated using TRIzol, and first-strand cDNA was synthesized from high-quality RNA using the HiScript III RT Super Mix kit for qPCR (including gDNA removal; Vazyme, Nanjing, China). qRT-PCR was conducted on a LightCycler 480 platform (Roche, San Diego, CA, USA) using ChamQ Universal SYBR qPCR Master Mix (Vazyme Biotech Co., Ltd., Nanjing, China) and gene-specific primer sets. *GmUNK1* (*Glyma.12g020500*) was the internal reference gene. Analyses included three technical replicates, and three independent biological replicates were analyzed per soybean genotype. The stability of *GmUNK1* expression was verified using NormFinder, yielding an M-value < 0.5. Relative transcript abundance was calculated using the 2^−ΔΔCt^ method. Results were analyzed via paired *t*-tests (α = 0.05) with Benjamini–Hochberg correction for multiple comparisons. Data are given as mean fold change ± 95% confidence interval [37].

### 4.4. Identification of Soybean RD22 Genes

Soybean genome sequences and corresponding protein datasets were obtained from the Phytozome v12.1 database. Candidate *RD22* genes were identified through BLASTP searches using conserved BURP domain-containing protein sequences as queries. Each candidate was further validated for the presence of the BURP domain (PF03181). Predicted subcellular localization of soybean RD22 proteins was determined using the Wolf PSORT online tool (version 0.2, https://wolfpsort.hgc.jp/). Protein physicochemical properties, including molecular weight, isoelectric point, and sequence length, were calculated using the ExPASy toolkit (https://web.expasy.org/protparam/).

### 4.5. Phylogenetic Tree Analysis

A phylogenetic tree was constructed using MEGA 10.0 (https://www.megasoftware.net/) based on amino acid sequences of RD22 homologs from multiple species, including soybean (Gm*RD22*), *Arabidopsis* (*AtRD22*), *Medicago truncatula* (*MtRD22*), *Brachypodium distachyon* (*BdRD22*), common bean (*PvRD22*), maize (*ZmRD22*), sorghum (*SbRD22*), and rice (*OsRD22*) [25]. The neighbor-joining (NJ) method was adopted for tree construction, and bootstrap analysis with 1000 replicates was performed to evaluate tree reliability.

### 4.6. Promoter Cis-Acting Element Analysis of GmRD22 Genes

To investigate transcriptional regulatory features, 2500 bp upstream sequences from the transcription start site of each *GmRD22* gene were extracted and analyzed for cis-acting elements using the PlantCARE database.

### 4.7. Analyses of GmRD22 Expression Patterns

Transcriptomic datasets from the Plant Expression Database were used to analyze *GmRD22* expression patterns across multiple soybean tissues, including leaves, roots, seeds, pods, flowers, and nodules. Expression levels were visualized as a heatmap generated through hierarchical clustering [38].

### 4.8. Overexpression and Silencing of GmRD22-3 in Soybean Hairy Roots

To investigate gene function, overexpression and RNA interference constructs were generated. The coding sequence of *GmRD22-3* was cloned into the pSOY1 vector for overexpression and into the pBWA(V)BS-hpRNA vector to create a hairpin RNA interference construct, both driven by the CaMV 35S promoter. These constructs were introduced into soybean hypocotyls using *A. rhizogenes* strain K599. Hairy roots transformed with the empty K599 vector served as controls. Transgenic roots were verified by PCR, and confirmed positive roots were isolated for further analysis. To evaluate the impact on SNF, nodules were collected 28 days after inoculation with the rhizobium strain HH103. Phenotypic measurements included nodule number, nodule dry weight, root dry weight, leghemoglobin content, and nitrogenase activity [39].

### 4.9. Physiological Observations

For internal color assessment, nodules were sectioned longitudinally and immediately assessed under a Leica MZ10F stereomicroscope (Leica Microsystems, Wetzlar, Hesse, Germany) under standardized imaging conditions. The content of leghemoglobin was quantified following established methods [40,41]. Briefly, approximately 1 g of fresh nodules was homogenized in 0.1 M phosphate buffer (pH 6.4) at 4 °C, followed by centrifugation (12,000× *g*, 20 min). Absorbances of the supernatants were read at 520, 540, and 560 nm using PBS as the blank. For transmission electron microscopy (TEM) (Thermo Fisher Scientific (FEI), Hillsboro, OR, USA), nodule segments (2 mm) were vacuum-fixed in 2.5% glutaraldehyde (Servicebio, G1102) (Servicebio Technology Co., Ltd., Wuhan, China) for 10 min and kept at 4 °C. After washing with PBS, post-fixation in 1% osmium tetroxide for 4 h, and dehydration in an ethanol gradient, samples were embedded in EPON 812 resin, sectioned (70 nm) using a Leica UC7 ultramicrotome (Leica Microsystems, Wetzlar, Hesse, Germany), and evaluated and imaged using an FEI Tecnai G2 Spirit BioTWIN TEM (Thermo Fisher Scientific (FEI), Hillsboro, OR, USA) at 100 kV. For RNA in situ hybridization, nodules collected at 28 days post-inoculation were fixed at 4 °C in RNase-free fixative containing 5.7% formaldehyde, 50% ethanol, and 5% acetic acid. A 130 bp fragment of *GmRD22-3* cDNA containing T7 and SP6 promoter sequences was amplified and cloned into the pSPT18 T-Easy vector (Roche (Roche Diagnostics GmbH, Mannheim, Baden-Württemberg, Germany), 11175025910). Digoxigenin-labeled sense and antisense RNA probes were generated using a DIG RNA labeling kit (Roche (Roche Diagnostics GmbH, Mannheim, Baden-Württemberg, Germany), 11175025910). Hybridization was visualized via DAB staining, and images were captured with a Leica DVM6 stereomicroscope (Leica Microsystems, Wetzlar, Hesse, Germany).

### 4.10. Haplotype Analysis

Haplotype analysis was performed on resequencing data from 3240 soybean accessions [42]. Sequence variation was examined across a 3 kb promoter region and the full genomic sequence of *GmRD22-3*. Variant filtering was performed using VCFtools (v0.1.17), retaining variants with a minor allele frequency greater than 5% and missing data below 10%. After removing non-informative and weakly associated variants, a set of 65 high-confidence SNPs was obtained. Haplotypes were classified using the geneHapR package (v1.2.5) in R, grouping accessions into three major haplotypes based on shared polymorphisms. A haplotype network was constructed and visualized using the get_hapNet and plotHapNet functions, with phylogenetic relationships being inferred via the pegas:haploNet module [43].

### 4.11. Statistical Analysis

All data were analyzed using GraphPad Prism (v10.4.0). Conventional molecular and gene expression experiments were performed with at least three independent biological replicates, whereas nodulation phenotypic tests were set with more than 15 biological replicates; the exact sample size (n) for each assay is specified in corresponding figure legends. Data were derived from at least three independent biological replicates. Differences between groups were evaluated using t-tests or Tukey’s multiple comparison tests, as appropriate. *p* < 0.05 was considered significant. Sequence analysis and visualization were conducted using TBtools (v1.132) [25].

### 4.12. Subcellular Localization

The subcellular localization of *GmRD22-3* was analyzed using a *Nicotiana benthamiana* transient expression system. The constructed fusion expression vector was infiltrated into tobacco leaf epidermal cells. After dark cultivation, GFP fluorescence signals were observed and photographed using a laser confocal scanning microscope, with a nuclear membrane marker used as a subcellular localization reference.

## 5. Conclusions

This study identified the soybean *GmRD22* family and characterized *GmRD22-3* as a negative regulator of SNF. Overexpression of *GmRD22-3* suppressed nodulation and nitrogenase activity, while silencing enhanced SNF, expanding the known functions of *RD22* genes. Haplotype analysis revealed strong artificial selection during domestication: the dominant haplotype HapI increased in frequency from wild to cultivated soybeans, showed a latitudinal gradient, and negatively correlated with soil nitrogen, suggesting an adaptive advantage under low-nitrogen conditions.

These findings provide new insights for marker-assisted and pyramiding breeding aimed at improving nitrogen use efficiency in soybean. Future studies will focus on dissecting the molecular regulatory network of *GmRD22-3* and its interactions with other SNF-related genes to further advance the theoretical framework governing SNF regulation in soybean.

## Figures and Tables

**Table 1:** Features of the 20 soybean *GmRD22* family genes.

Gene Name	Gene ID	Gene Location	CDS Length (bp)	Protein Length (aa)	Isoelectric Point	Molecular Weight (kDa)	Subcellular Localization
*GmRD22-1*	*Glyma.04G079600*	Gm46629336-6633386	1026	341	6.94	36.99	Chloroplast
*GmRD22-2*	*Glyma.01G030900*	Gm13236937-3240378	1890	629	8.75	68.20	Chloroplast
*GmRD22-3*	*Glyma.04G013500*	Gm41026559-1028565	1041	346	6.78	39.28	Chloroplast
*GmRD22-4*	*Glyma.12G217400*	Gm1239147312-39150346	831	276	5.80	30.88	Chloroplast
*GmRD22-5*	*Glyma.18G138200*	Gm1820430574-20433040	1671	556	7.01	60.88	Plastid
*GmRD22-6*	*Glyma.06G081100*	Gm66189125-6193027	1032	343	7.69	37.21	Chloroplast
*GmRD22-7*	*Glyma.06G013400*	Gm6990489-992108	1053	350	5.89	39.78	Chloroplast
*GmRD22-8*	*Glyma.08G287500*	Gm839280742-39283173	1184	627	8.38	68.32	Chloroplast
*GmRD22-9*	*Glyma.04G180400*	Gm443444987-43450251	1662	553	7.23	62.42	Chloroplast
*GmRD22-10*	*Glyma.12G217300*	Gm1239141473-39143391	819	272	6.36	30.42	Chloroplast
*GmRD22-11*	*Glyma.06G183400*	Gm615731051-15734210	1890	629	8.92	69.26	Chloroplast
*GmRD22-12*	*Glyma.14G140900*	Gm1423588819-23591976	1050	349	6.05	37.98	Chloroplast
*GmRD22-13*	*Glyma.08G036600*	Gm82887849-2890875	498	165	8.21	18.34	Chloroplast
*GmRD22-14*	*Glyma.12G044600*	Gm123225606-3228857	1626	541	5.98	59.69	Chloroplast
*GmRD22-15*	*Glyma.11G119900*	Gm119125316-9128958	1716	571	6.91	63.93	Chloroplast
*GmRD22-16*	*Glyma.02G034700*	Gm23191535-3194757	1887	628	8.31	68.31	Chloroplast
*GmRD22-17*	*Glyma.04G182300*	Gm443668804-43672025	1896	631	9.03	69.31	Chloroplast
*GmRD22-18*	*Glyma.18G138000*	Gm1820266114-20268505	1869	622	6.21	67.87	Chloroplast
*GmRD22-19*	*Glyma.14G141000*	Gm1423609295-23612611	1104	367	8.54	39.62	Chloroplast
*GmRD22-20*	*Glyma.11G119100*	Gm119033245-9034638	900	299	7.00	33.88	Chloroplast

## Data Availability

The original contributions presented in this study are included in the article/Supplementary Material. Further inquiries can be directed to the corresponding authors.

## Supplementary Materials

The following supporting information can be downloaded at https://www.mdpi.com/article/10.3390/plants15152276/s1: Figure S1: GO and KEGG enrichment analysis of differentially expressed genes. Figure S2: Temporal expression dynamics of *GmRD22* family genes during soybean nodule development. Figure S3: Subcellular localization of *GmRD22-3* in Nicotiana benthamiana epidermal cells. Figure S4: Evolutionary relationships and synteny analysis of the *GmRD22* gene family. Figure S5: Functional validation and population genetic analysis of *GmRD22-3*.
